# Controlled growth of 1D and 2D ZnO nanostructures on 4H-SiC using Au catalyst

**DOI:** 10.1186/1556-276X-9-379

**Published:** 2014-08-03

**Authors:** Abhishek Singh Dahiya, Charles Opoku, Daniel Alquier, Guylaine Poulin-Vittrant, Frederic Cayrel, Olivier Graton, Louis-Pascal Tran Huu Hue, Nicolas Camara

**Affiliations:** 1 Université François Rabelais de Tours, CNRS, GREMAN UMR 7347, 16 rue Pierre et Marie Curie, Tours 37071, France

**Keywords:** Zinc oxide, Nanostructures, Nanowires, Nanowalls, Zinc cluster drift

## Abstract

A perfect control of nanostructure growth is a prerequisite for the development of electronic and optoelectronic device/systems. In this article, we demonstrate the growth of various ZnO-derived nanostructures, including well-ordered arrays of high aspect ratio single crystalline nanowires with preferred growth direction along the [0001] axis, nanowalls, and hybrid nanowire-nanowall structures. The growths of the various ZnO nanostructures have been carried out on SiC substrates in a horizontal furnace, using Au thin film as catalyst. From experimental observations, we have ascribed the growth mechanisms of the different ZnO nanostructures to be a combination of catalytic-assisted and non-catalytic-assisted vapor–liquid-solid (VLS) processes. We have also found that the different ZnO nanoarchitectures' material evolution is governed by a Zn cluster drift effects on the SiC surface mainly driven by growth temperature. Au thin film thickness, growth time, and temperature are the parameters to optimize in order to obtain the different ZnO nanoarchitectures.

## Background

Extensive research efforts have been recently dedicated to the synthesis of high-quality zinc oxide (ZnO) nanostructures, targeting high-performance electronic and optoelectronic applications [1-6]. Devices such as field-effect transistors [1], sensors [2], field emission [3] photovoltaic [4], room temperature UV lasers [5], and light-emitting diodes [6] have already been investigated in the literature. The interest in ZnO nanomaterials has been largely driven by the material's excellent electrical and optoelectronic properties, including direct wide band-gap (3.37 eV), high exciton binding energy (60 meV), and moderate to high electron mobility (1 to 200 cm^2^/Vs) [1,4]. Moreover, ZnO's excellent piezoelectric and pyroelectric properties are finding widespread applications targeting various energy harvesting systems [7-11].

Synthesis strategies, including carbothermal reduction [12-22], pulse laser deposition [23], and hydrothermal [24] and electrochemical deposition [25], have been widely exploited for growing ZnO nanostructures such as nanowires (NWs), nanowalls (NWLs), and/or a hybrid of the two aforementioned nanostructures. Among them, carbothermal reduction of ZnO powder is offering high-quality ZnO nanostructures via the VLS process. In this process, a so-called seed thin layer of metal (such as Au) is first deposited onto the desired substrate. When increasing the temperature, the catalyst seed layer of metal is converted into nanoparticles. The nanoparticles can act as sink sites for vapors of the desired nanomaterial. In some cases, the vapors are efficiently trapped by the metal catalyst islands, and during the growth (‘tip’ growth), the metallic nanoparticle rides atop the nanostructures. In some others, the metal nanoparticle acts only as the nucleation site and not as a catalyst for nanomaterial growth. In this case, the metal nanoparticles remain at the bottom of the nanomaterial during growth (‘base’ growth) [10,15-17,21]. In addition to this ‘base’ growth, one may also observe side branches growing from the bottom of the nanostructures. The latter scenario often results in the formation of complete nanostructured networks such as nanowalls (NWLs) [19]. Such structures are quasi-2D nanomaterials with potential applications in emerging technologies, including solar cells [26], sensors [23,27], and piezoelectric nanogenerators [10]. It has been shown that NWs and NWLs can also co-exist in a single synthesis batch [15]. Kumar et al. [10] successfully demonstrated the growth of NWs, NWLs, and hybrid nanowire-nanowall (NW-NWL) in which material morphology was optimized by careful control of the metal layer (Au) thickness. On the other hand, some reports have shown that various ZnO nanostructures can also be produced through precise control of the temperature-activated Zn source flux during a vapor transport and condensation synthesis process [15]. Despite these several reports of different ZnO nanostructure growth processes, the exact mechanism responsible for the evolution of the different nanostructures is still not fully understood.

In this paper, we will present a detailed study of the growth and evolution of a diverse range of ZnO nanostructures that can be grown on Au-coated 4H-SiC substrates. We will emphasize that VLS synthesis and its optimization is driven by Au layer thickness, growth temperature, and time. Finally, we will demonstrate that the diverse nanostructures obtained here can be attributed to the temperature-activated Zn cluster drift phenomenon on the SiC surface and, hence, can be controlled.

## Methods

### Experimental details

The synthesis of the different ZnO nanostructures was carried out in a horizontal quartz furnace [14,21]. ZnO nanostructures were grown by carbothermal reduction of ZnO nanopowder [21] on (0001) 4H-SiC substrates. SiC was chosen to target a crystalline vertically oriented ZnO growth keeping the lattice mismatch as small as possible (<6 %). Indeed, it has been recently shown that, for energy harvesting applications, vertically c-axis oriented nanostructures such as NWs and NWLs are preferred over randomly oriented ones [7,8,10,11]. Prior to nanomaterial synthesis, SiC substrates were coated with two different Au thicknesses (6 and 12 nm ±1 nm) using a magnetron sputtering system. Next, the Au-coated SiC substrates and the source material (ZnO and C at 1:1 weight ratio) were placed on top of an Alumina ‘boat.’ This boat was inserted close to the center of quartz tube inside the furnace. During all the process, an Ar ambient was maintained in the growth chamber, without any vacuum system. In this work, the growth temperature was varied from 850 to 900°C with a ramp rate of 30°C min^-1^ up to the dwell temperature while the growth time at the plateau ranged from 10 to 180 min. After the growth, the furnace was switched off and left to cool down naturally to room temperature. Samples were then removed from the growth chamber and characterized. Details of experimental parameters and the resulting nanomorphologies are summarized in Table 1, while Au nanoparticle density and mean radius are presented in Table 2.

**Table 1:** Growth parameters for various ZnO nanostructures

**S. No**	***m***_**source**_**, ZnO/C (ratio)**	**Au thickness (nm)**	**Temperature of growth (°C)**	**Ar flow (sccm)**	**Time of growth (min)**	**Resulting morphology**
1	1:1	6	850	700	90	High-density nanowires
2	1:1	12	850	700	10	Low-density nanowires
3	1:1	6	850	700	10	High-density nanowires
3	1:1	6	900	700	90	Nanowire-nanowall hybrid
4	1:1	6	900	700	180	Nanowall
5	1:1	12	900	700	90	Nanowire-Zn cluster drift hybrid
6	1:1	12	900	700	180	Nanowire-nanofin hybrid

**Table 2:** Density and mean radius of Au nanoparticles and ZnO NWs

	**Au layer thickness (nm)**	**Density (/μm**^**2**^**)**	**Mean radius (nm)**	**Temperature of annealing/growth (°C)**
Au nanoparticle	12 ± 1.5	5 ± 1	69 ± 31	800
		5 ± 1	151 ± 71	700
		5 ± 1	207 ± 114	600
	6 ± 1	125 ± 10	21 ± 7	800
		125 ± 10	25 ± 10	700
		125 ± 10	28 ± 12	600
ZnO nanowire	12	5 ± 1	42 ± 15	850
	6	70 ± 10	35 ± 15	850

Results of the growths have been characterized in three different equipments. First, a dual beam FEI Strata 400 (FEI, Hillsboro, OR, USA), a focused ion beam (FIB) coupled to a scanning electron microscopy (SEM) system, has been used. It is equipped with a flip stage, a scanning transmission electron microscopy (STEM) detector, and an energy-dispersive X-ray spectroscopy (EDX) for sample transfer, observation, and elemental composition characterization, accordingly. Additionally, NW and NWL lamellas have been prepared using the FIB mode and then characterized in STEM mode, but also in a second equipment: a high-resolution transmission electron microscopy (HRTEM) using a JEOL 2100 F (JEOL Ltd., Akishima-shi, Japan) operating at an accelerating voltage of 200 kV. Finally, the ZnO nanostructure crystallinity was studied using X-ray diffraction (XRD) with CuK*α*_1_ radiation on the high resolution parallel beam diffractometer Bruker D8 discover (Bruker AXS, Inc., Madison, WI, USA). The scans were performed in the 2*θ* range from 25° to 85° at a scanning rate of 0*.*01° s^-1^.

## Results and discussion

**Figure 1:**
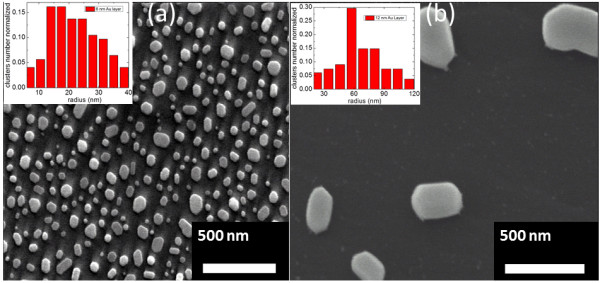
SEM images of (a) 6-nm and (b) 12-nm ‘seed layer’ Au thin film annealed at 800°C on SiC substrate.

**Figure 2:**
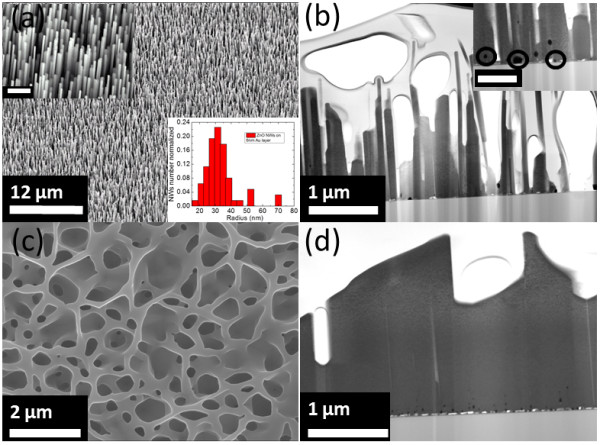
**Typical SEM and STEM ZnO nanoarchitectures images. (a)** 22° side-view SEM image of ZnO NWs. Inset shows the high magnification of the sample. Scale bar is 1 μm. **(b)** Corresponding STEM image of the sample. Inset shows the high magnification of the sample showing the presence of Au nanoparticles at the ZnO/SiC interface. Scale bar is 500 nm. **(c)** Top-view SEM image of ZnO NWLs. **(d)** Corresponding STEM image of the sample.

**Figure 3:**
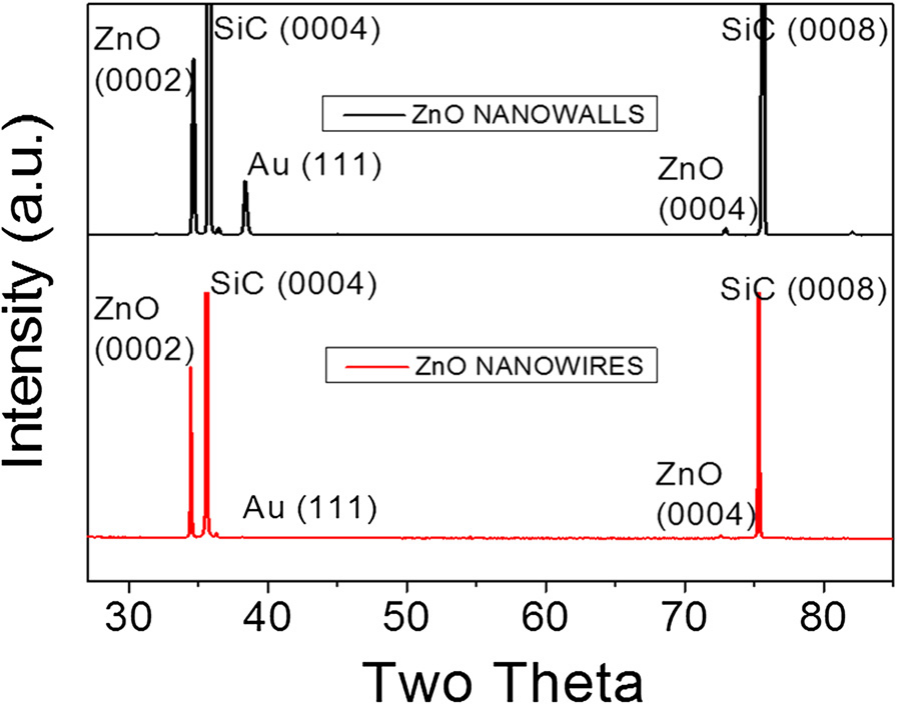
XRD patterns of ZnO nanowalls and nanowires.

**Figure 4:**
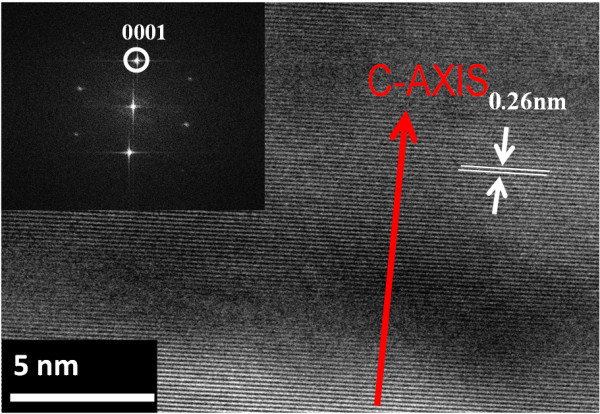
HRTEM image of ZnO NW including the selected area diffraction pattern as inset.

**Figure 5:**
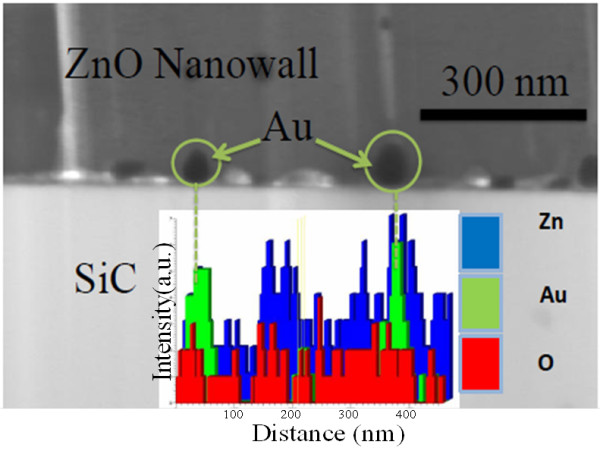
High-magnification STEM image of ZnO NWLs and the area scanned for EDX analysis.

It has been shown, in the literature, that the starting Au seed layer thickness can significantly influence the final outcome of the nanostructures [10,12,15]. The nanostructures, in this work, have been grown on Au-coated hexagonal SiC surfaces. During the temperature ramp, from approximately 400°C, the Au film is found to efficiently transform into islands of Au droplets. In addition to this, the clusterization of the Au layer is expected to follow the ripening process during the early stages of synthesis. As discussed by Ruffino et al*.*[28], the ripening process results in the formation of 3D nanostructures, due to the thermally activated surface diffusion of Au atoms. To gain detailed understanding of both the seed layer clustering and subsequent ZnO nanostructure formation, it was important to understand the clusterization processes exhibited by different Au layer thicknesses: in our experiment, 6 and 12 nm. To follow the change in Au layer morphology and to evaluate the size distribution of Au nanoparticles, SEM images were assessed. Figure 1 shows typical SEM images of the nanoparticles obtained for the different Au layer thicknesses followed by thermal annealing at 800°C in Ar ambient without ZnO growth precursors. For both thicknesses, the Au films were effectively converted into uniformly distributed spherical and/or hexagonal-like nanoparticles. This behavior can be explained by the non-wetting characteristics between Au and SiC substrate interface. Notably, with increasing Au film thickness from 6 to 12 nm, the coverage density of Au nanoparticles were found to decrease from around 130 μm^-2^ (Figure 1a) to 5 μm^-2^ (Figure 1b), respectively. As expected, the thickness of the initial Au layer strongly affects the density of the Au nanoparticles and, hence, as shown later in this work, the density of the resulting ZnO nanostructures produced. The insets in Figure 1a, b show the Au cluster size distribution for the Au layer thickness of 6 and 12 nm, respectively annealed at 800°C for 30 min in Ar ambient.Based on these observations, we first carried out the growth on the 6-nm Au seed layer samples. In Figure 2a, b, typical SEM and STEM images of ZnO NWs grown at 850°C for 90 min are presented. From Figure 2a, b, it can be seen that a high-density NW with an exceptional degree of material orientation perpendicular to the SiC substrate is achieved. From the SEM and STEM images, typical NW length and diameter were determined to be around 1 to 2 μm and 30 to 140 nm, respectively (longer nanowires can be obtained simply by increasing the growth time). Based on the nanowire length and growth time, the growth rate for the present NWs was determined to be approximately 15 to 20 nm/min. Figure 2c,d shows typical SEM and STEM images of vertically oriented ZnO NWLs grown at 900°C for 180 min. From Figure 2c, d, it is noticeable that the measured height and widths of the NWLs were also found to be consistent with those measured for the NWs, thus suggesting a similar growth process for both types of nanostructures.

In Figure 3, we present the XRD patterns exhibited by the ZnO NWs and NWLs. These XRD patterns suggest that both NWs and NWLs are highly crystalline wurtzite ZnO. Indeed, the 2*θ* peaks appearing at 34.42° and 72.5° correspond to the [0002] and [0004] directions, consistent with a growth along the c-axis of hexagonal ZnO. Moreover, the excellent material crystallinity, found by the XRD measurements, suggests that the present nanomaterials are potentially valuable for high-performance ZnO-based nanosensor and nanoactuator applications. The other peaks appearing at 35.7°, 75.6°, and 38.18° in Figure 3 correspond to single crystalline [0002] and [0004] directions of the SiC substrate and the Au (111) catalyst, respectively. To confirm these results, HRTEM analysis were also carried out on individual ZnO NWs. A representative HRTEM image can be found in Figure 4. First, the electron diffraction pattern of the ZnO NW confirms the high crystallinity of the material. Moreover, the distance between adjacent planes (lattice fringes) along the NW length was measured to be 0.26 nm, consistent with that of (0001) wurtzite ZnO phase.

As mentioned previously, in the VLS process, the location of metal catalyst after the growth is essential for the determination of the growth process. To determine the exact position of the Au nanoparticles, EDX experiments were carried out on both NWs and NWLs. Figure 5 shows an example of high-magnification cross-section STEM image of ZnO NWLs and the area scan used for the EDX analysis. From this figure, it can be seen that the Au nanoparticles are located close to the ZnO-SiC interface. The presence of Au nanoparticle at the ZnO/substrate interface is well documented in the literature [10,15-17,21]. However, the exact mechanism responsible for the growth process of such diverse nanostructures is not fully understood. The observation of the Au seed particle at the ZnO/substrate interface would suggest that the growth of the nanostructures is due to the non-catalytic-assisted VLS. However, we will show in later sections that the apparent location of the Au seed particles can also be due to a combination of catalytic-assisted and non-catalytic-assisted VLS processes [15].

**Figure 6:**
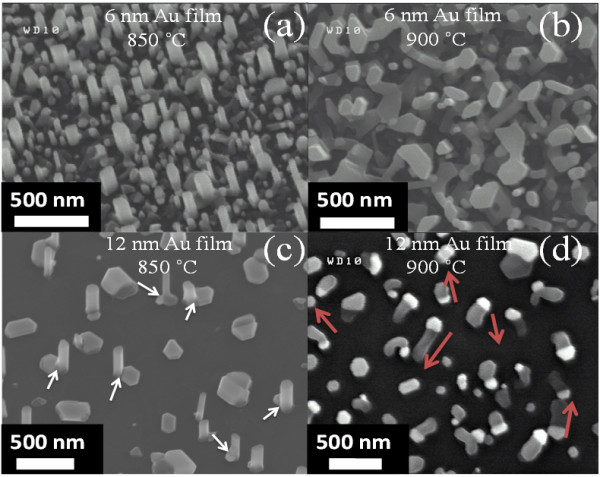
**SEM images of ZnO NWs and Zn cluster drift phenomenon.** SEM images of ZnO NWs grown for 10 min on high density of Au nanoparticles at **(a)** 850°C and **(b)** 900°C or on low density of Au nanoparticles at **(c)** 850°C and **(d)** 900°C. As pointed out by the arrows in **(c)**, the ZnO NWs appear to protrude from the edges of the Au nanoparticles, while the arrows in **(d)** show the random motion of Zn cluster drift.

**Figure 7:**
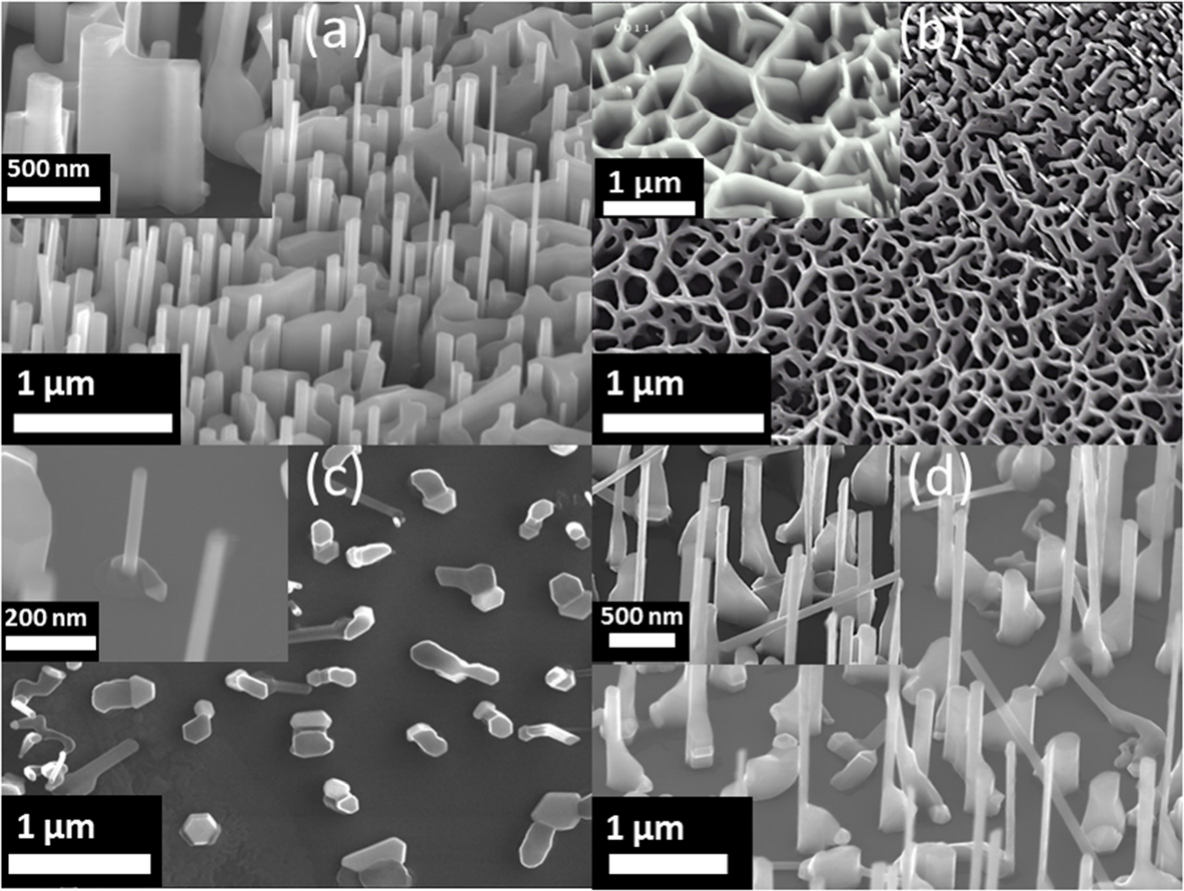
**SEM images of NW-NWL hybrid, ZnO NWL, and nanofin-NW hybrid. (a)** Low magnification 52° side-view SEM image of the NW-NWL hybrid. Inset, higher magnification 52° SEM image shows the formation of NWL. Scale bar is 500 nm. **(b)** Top-view SEM image of ZnO NWL. Inset, higher magnification 52° side-view SEM image of the sample. Scale bar is 1 μm. **(c)** Top-view SEM image shows the presence of Au catalyst at the root and Zn cluster drift in random directions terminated with growth of NW. Inset shows higher magnification 52° side-view SEM image of the sample. Scale bar is 200 nm. **(d)** Low magnification 52° side-view SEM image of the nanofin-NW hybrid. Inset shows higher magnification 52° side-view SEM image of the sample. Scale bar is 500 nm.

To gain a better understanding of the growth processes/mechanisms responsible for the formation of the various ZnO nanostructures, the early stages of material synthesis are crucial. Hence, as presented in Figure 6, we have examined nanostructure growth processes varying the main synthesis parameters, i.e., Au layer thicknesses and temperature, keeping all the other parameters, such as time (10 min), constant. Figure 6a, b shows, respectively, the SEM images of the resulting ZnO nanostructures grown at 850 and 900°C on the high-density Au nanoparticle sample (6 nm Au). From Figure 6a, at 850°C, the resulting ZnO nanostructures resemble NW formation (see also Figure 2a, b), while at 900°C, in Figure 6b, it can be seen that a complete nanostructured network formation has been started. However, the nanostructure density, in such samples, makes it difficult to elucidate the exact growth mechanism. Further, similar experiments were carried out on samples exhibiting low density of Au nanoparticles (12 nm Au). Figure 6c, d shows the SEM images of the resulting ZnO nanostructures grown at 850 and 900°C, respectively. At 850°C, the ZnO NWs appear to protrude from the edges of the Au nanoparticles, as pointed out by arrows in Figure 6c. For the sample grown at 900°C, one can note that Zn clusters appear to drift significantly, with no preferential direction, as indicated by the arrows in Figure 6d. It is important to mention that this behavior was absent at 850°C, leading only to NW growth. Using a similar synthesis approach, Shi et al. [19] have demonstrated the random motion of Zn cluster drift effects above 700°C during the synthesis of ZnO nanostructures (nanowires, nanofins, and hybrid nanowire-nanofins) on gallium nitride (GaN) substrate. The authors then used thermally activated Brownian motion of the Zn clusters to explain the evolution of their NWLs. The major difference between their work and the present investigations is the temperature of Zn drift. Such a disparity in temperature-activated Zn cluster drift may be related to the fact that their growth was performed at comparatively lower pressure (20 Torr), without any metal catalyst (Au in our case). As the Zn clusters were not attached to any seed particles, the probability of Zn cluster drift on the surface is expected to be higher at comparatively lower temperature. However, one can notice that the length of the drift appeared to be influenced by the synthesis temperature, similar to observations in [19]. Indeed, at 850°C (Figure 6a, b, c), we observed a negligible drift, while, at 900°C, the length of the drift was found to vary from 100 to 400 nm. In the case of the high-density Au nanoparticles on SiC substrate, the average distance between neighboring Au nanoparticles was measured to be less than 200 nm. Hence, at 900°C, the drift phenomenon is effectively halted when a Zn cluster encounters another Zn cluster trace or a Au nanoparticle, as mentioned in [19]. This in turn resulted in the formation of interconnected networks of ZnO, as shown in Figure 6b. This is the exact observation that can be made in Figure 7b, where NWLs are obtained on high Au particle densities and at comparatively higher growth temperatures (900°C), as a result of the Zn clusters coalescing.

To follow the morphological evolution of the ZnO nanostructures, time-dependent growths were also carried out on the SiC substrates using the different Au nanoparticle densities. For this present investigation, the growth temperature was fixed at 900°C, while the growth times were either 90 or 180 min. Figure 7 presents the experimental results obtained for ZnO nanomaterial synthesis as a function of time. In Figure 7a, b, the growth of the ZnO NW-NWL hybrids and NWLs is obtained by varying time between 90 and 180 min, respectively, for the high-density Au nanoparticle case. Once again, the drifting was effectively halted by Zn clusters merging with other clusters and/or Au seed nanoparticles resulting in the formation of complete ZnO networks over large areas of the SiC substrates, as already shown in Figure 6b. When growing with low-density Au nanoparticles, the following observations can be made: (i) the drift of the Zn cluster results in the formation of vertically oriented ZnO NWs at the Zn cluster drift sites and not at the seed particle site as shown in Figure 7c, and (ii) with increasing growth time (Figure 7d), a new form of nanostructure can be observed, in which NWLs are effectively terminated by NWs at one end. These observations were found to be consistent with the so-called nanofins, reported in [19]. With longer synthesis time (180 min), we observed that the boundaries between ZnO NWs and horizontal trace of the Zn cluster were more favorable nucleation sites, forcing the growth of the observed ZnO nanofin-NW structures.

**Figure 8:**
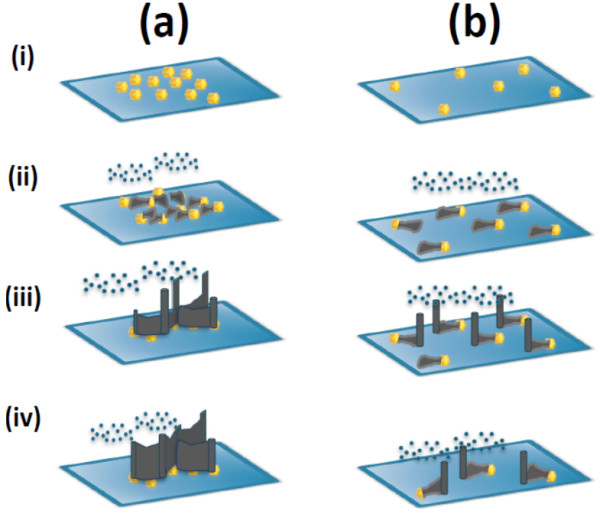
**Schematic of growth mechanism for ZnO nanoarchitectures.** Schematic of the growth mechanism for ZnO nanoarchitectures at 900°C with **(a)** high density of Au nanoparticles and **(b)** low density of Au nanoparticles.

Based on the experimental observations, the growth mechanisms for ZnO nanoarchitectures at 900°C are schematically illustrated in Figure 8. The first step of the process is the conversion of the Au thin film into spherical- and/or hexagonal-shaped nanoparticles, described by the ripening process [28]. The density of the Au nanoparticles, which can be controlled by the thickness of the sputtered Au layer, plays a key role in determining the final morphology of ZnO nanostructure. Increasing the Au film thickness from 6 to 12 nm also results in a decrease in the coverage densities of the Au nanoparticles, as illustrated in (i) in Figure 8a,b, respectively. At the desired growth temperature (900°C), carbothermally reduced Zn vapors are generated and efficiently captured by Au nanoparticles. The capturing processes occur on the Au droplets since Zn vapor trapping is energetically more favorable at these sites than at the SiC surface. The supply of Zn vapors is expected to either condense directly into the Au nanoparticle or be transported from adjacent regions on the growth substrate into the Au droplets/clusters to form clusters of Au-Zn alloys. The eutectic temperature of Au-Zn systems was estimated to be around 683°C [29] with a Zn maximum solubility in Au of 33.5 at%. However, throughout this present investigation, the growth temperatures (850 or 900°C) were well above the eutectic temperature for Au-Zn systems. As such, Au and Zn can be expected to be molten alloy droplets on the substrates. The formation of such droplets can be well described by the following expression [30].

(1) $$\mathrm{Zn}\left(v\right)+\mathrm{Au}\left(s\right)\to \mathrm{Au}-\mathrm{Zn}\left(l\right)$$

With increasing growth time, the continual supply of Zn vapors results in an increase in Zn concentration in Au-Zn alloy clusters. The process of Zn condensation/dissolution within the Au-Zn alloy system continues until the supersaturation point, where a solid crystal of ZnO nucleates out of the molten alloy droplet [30]. However, the present experimental work shows that depending on the system (growth) temperature, ZnO nucleation can occur either on the Au-Zn alloy droplets (850°C, Figure 6c) or away from the Au-Zn alloy droplet (900°C). At 900°C, Zn-rich clusters that are precipitated on Au-Zn alloy droplets experience a drift as a result of the high thermal energy [19]. In our system, it was observed that at 700 sccm of Ar flow, the Zn cluster drift phenomenon can be significant above 850°C. As can be seen in Figure 8b (ii), the Zn cluster appears to drift with no preferential direction. The Zn cluster drift was subsequently halted either by (1) merging with other moving Zn cluster traces and/or Au-Zn alloy droplets (Figure 8a (ii) for the high density of Au nanoparticle case), (2) sticking on a substrate defect site, and/or (3) reduction in the local substrate temperature (Figure 8b (ii) for the low density of Au nanoparticle case). With continual supply of Zn vapors and residual oxygen atoms inside the growth chamber, precipitation of ZnO NWs via self-catalyzed VLS process is established (Figure 8 (iii)). Beyond this stage, NW growth is effectively controlled by a non-catalytic-assisted VLS mechanism and the Au nanoparticles play no further role in the evolution of the growth process [16,22]. In the final step of growth, as the edges between the NW and drifted traces are thermodynamically more favorable sink sites for incoming Zn vapors, ZnO NWL networks are formed. This is clearly demonstrated in the case of high-density Au nanoparticles, as shown in Figure 8a (iv). On the other hand, when the distance between the Au nanoparticles is significantly larger than the drifted Zn length, as in the low-density case, the growth process can also result in the formation of NW-nanofin hybrid structures with prolonged synthesis time (as depicted in Figure 8b (iv)).

## Conclusions

In summary, controlled growth of various ZnO nanostructures, including nanowires (NWs), nanowalls (NWLs), and hybrid nanowire-nanowall, was demonstrated through careful control of key experimental parameters, including Au seed thickness, synthesis temperature, and time, via a combination of catalytic-assisted and non-catalytic-assisted VLS processes. A combination of nanomaterial characterization techniques revealed that highly crystalline wurtzite nanostructures were produced. Experimental work presented here suggests that the nanomaterial synthesis temperature effectively controlled the Zn cluster drift phenomenon, responsible for the formation of the various studied ZnO nanostructures. NWs were found to grow at comparatively lower temperatures, and the overall NW density was effectively controlled through the Au seed film thickness. High-density Au clusters and high growth temperatures resulted in NWLs and hybrid NW-NWL formation. The formation of such structures was found also to depend on the synthesis time. These results offer a new prospective towards the development of applications that require various predefined ZnO nanostructures on [0001]-oriented SiC as well as other similar compound substrates, including GaN, AlN, and GaN-on-Si substrates targeting future high-performance nanodevices.

## Competing interests

The authors declare that they have no competing interests.

## Authors’ contributions

ASD and NC designed the experiments. ASD also performed the synthesis of the various ZnO nanostructures and majority of structural/morphological analysis of the nanostructures. FC prepared the TEM lamellas and performed HRTEM and EDX characterization for the different ZnO nanostructures. The drafting of the manuscript has been done by ASD, OG, and CO. DA carried out the gold annealing studies. DA, LPTHH, GPV, and NC did critical revisions of the manuscript. All authors have read and approved the final manuscript.
